# Evaluating the Effect of Vitamin D Supplementation on Type 2 Diabetes Risk: A Systematic Review

**DOI:** 10.7759/cureus.75860

**Published:** 2024-12-17

**Authors:** Ahmed Altayeb Abbas Fadlallah, Miska Haroun Mohamed Hassan, Salma Farah, Solar Eltayeb Mohamed Gaffar, Ashraf Hayder Mahgoub Ali, Hasna Salem Alzahrani, Amirah Hassan Alhadhrami, Fahad Salem M Alqahtani

**Affiliations:** 1 Faculty of Medicine, University of Khartoum, Khartoum, SDN; 2 General Practice, Sukoon International Extended Center, Jeddah, SAU; 3 Palliative Care Medicine, University Hospital Galway, Galway, IRL; 4 Intensive Care Unit, Dr. Sulaiman Al Habib Hospital, Buraidah, SAU; 5 Cardiology, Al Qassimi Hospital, Sharjah, ARE; 6 Social Work, Najran Armed Forces Hospital, Najran, SAU; 7 Quality and Patient Safety, Najran Armed Forces Hospital, Najran, SAU; 8 Family Medicine, Najran Armed Forces Hospital, Najran, SAU

**Keywords:** 25(oh)d, prediabetes, systematic review, type 2 diabetes, vitamin d

## Abstract

Although observational studies have linked vitamin D deficiency to diabetes, it is unknown if taking vitamin D supplements can reduce the chance of developing type 2 diabetes mellitus (T2DM). The purpose of this systematic review is to determine whether vitamin D supplementation lowers the risk of type 2 diabetes. The Preferred Reporting Items for Systematic Reviews and Meta-Analyses (PRISMA) guidelines were used to search for studies based on pre-established inclusion and exclusion criteria. Five different databases (Scopus, Web of Science, PubMed, Google Scholar, and Cochrane Library) were used to search for relevant studies. These databases contained 1,916 relevant research papers that were examined for duplication using the EndNote software. After a careful text analysis, only 13 of these articles were found to be pertinent. The Newcastle-Ottawa Scale (NOS) was used to assess the risk of bias in each included study. Ten of the 13 trials examined how vitamin D supplementation affected the homeostasis model assessment of insulin resistance (HOMA-IR) of the patients. Patients using vitamin D supplements saw a drop in their HOMA-IR in six of these RCTs but an increase in four of them. The vitamin D group had a lower HOMA-IR than the placebo cohort in seven of the 10 trials that examined HOMA-IR. Despite the impact on insulin resistance, there is not enough data to conclude that vitamin D supplement significantly lowers the incidence of T2DM. To impact clinical guidelines about supplementation with vitamin D among people at risk of type 2 diabetes, more studies in this area might be beneficial.

## Introduction and background

There is a well-established observational link between diabetes and vitamin D insufficiency [1]. However, there is currently no solid evidence to support the claim that vitamin D therapy enhances glycemic control [2]. The possible role of vitamin D in glucose metabolism has been explained by a number of mechanisms, including (1) the direct stimulation of insulin production via the pancreatic beta cell's vitamin D receptor, (2) reduction of systemic inflammation and consequent improvement in insulin resistance, and (3) amelioration of peripheral insulin resistance through the liver and muscles' vitamin D receptors [3-5].

Numerous randomized controlled trials (RCTs) have been carried out in recent years to determine if vitamin D supplementation can enhance glycemic control in persons with type 2 diabetes mellitus (T2DM). The three recently published systematic reviews studying the effectiveness of vitamin D therapy in improving glycemic control in individuals with type 2 diabetes revealed no advantages, in contrast to observational research demonstrating the positive function of vitamin D in the metabolism of glucose [6-8]. A systematic review by Haroon et al. included both healthy and subjects with a fasting glucose level of type 2 diabetes; two recent reviews included investigations using different forms of vitamin D, such as intramuscular injections of vitamin D or active vitamin; however, these reviews were limited by clinical and approach heterogeneity in the included studies [7].

The majority of cross-sectional observational research has found a negative correlation between the frequency of hyperglycemia and vitamin D status. Higher 25(OH)D levels were also linked to a decreased incidence of diabetes, according to longitudinal research [9]. Furthermore, a correlation between low plasma 25(OH)D levels and an elevated risk of type 2 diabetes was noted in the study by Afzal S et al. [10]. However, a controlled experiment of vitamin D supplements should examine this causative link. Vitamin D supplementation's impact on prediabetes has recently been documented. Vitamin D supplementation has been linked in studies to better insulin sensitivity and beta cell activity in people at elevated risks for diabetes but not in people with normal baseline fasting glucose [11].

The impact of taking vitamin D supplements on individuals with type 2 diabetes has been assessed in several RCTs; however, the results of these studies have been mixed. In patients with diabetes type 2 or impaired glucose tolerance, a recent systematic analysis revealed weak correlations between vitamin D treatment, improved resistance to insulin, and decreased fasting glucose. However, the prior studies included patients with both type 2 diabetes and glucose intolerance, which resulted in heterogeneity. In addition, there were not many included studies. Consequently, this study's primary goal was to employ the meta-analysis process to thoroughly examine how vitamin D supplementation affected glycemic measurements in individuals with type 2 diabetes alone.

## Review

Methodology

This systematic review followed the recommendations of PRISMA guidelines (Preferred Reporting Items for Systemic Reviews and Meta-Analyses) [12].

Search Strategy

To find studies published in English, we look through five distinct databases without considering the publication date. Furthermore, we searched these databases for any ongoing or past systematic reviews on the topic. Using the EndNote software, results from five databases were merged, and duplicates were removed. A list of the databases and search methods used is provided in Table 1.

**Table 1:** Search strategies used for different databases *TITLE-ABS-KEY* is a search field in the Scopus database that allows users to search for information across titles, abstracts, and keywords. *TS* in Web of Science stands for "topic."

Sr. no.	Database	Search string
1	Scopus	TITLE-ABS-KEY(("vitamin D supplementation" OR "vitamin D intake" OR "vitamin D treatment") AND ("type 2 diabetes" OR "T2D" OR "type 2 diabetes mellitus" OR "diabetes mellitus type 2") AND ("risk" OR "prevention" OR "onset"))
2	Web of Science	TS=("vitamin D supplementation" OR "vitamin D intake" OR "vitamin D treatment") AND TS=("type 2 diabetes" OR "T2D" OR "type 2 diabetes mellitus" OR "diabetes mellitus type 2") AND TS=("risk" OR "prevention" OR "onset")
3	PubMed/EMBASE	("vitamin D supplementation"[Title/Abstract] OR "vitamin D intake" [Title/Abstract] OR "vitamin D treatment"[Title/Abstract]) AND ("type 2 diabetes"[Title/Abstract] OR "T2D"[Title/Abstract] OR "type 2 diabetes mellitus"[Title/Abstract] OR "diabetes mellitus type 2"[Title/Abstract]) AND ("risk"[Title/Abstract] OR "prevention"[Title/Abstract] OR “onset"[Title/Abstract])
4	Google Scholar	("vitamin D supplementation" OR "vitamin D intake" OR "vitamin D treatment") AND ("type 2 diabetes" OR "T2D" OR "type 2 diabetes mellitus" OR "diabetes mellitus type 2") AND ("risk" OR "prevention" OR "onset")
5	Cochrane Library	("vitamin D supplementation" OR "vitamin D intake" OR "vitamin D treatment") AND ("type 2 diabetes" OR "T2D" OR "type 2 diabetes mellitus" OR "diabetes mellitus type 2") AND ("risk" OR "prevention" OR "onset")

Study Selection

Every article was retrieved and saved in the EndNote library (ENDNOTE X9) after duplicates had been removed during the extraction process. Two separate reviewers selected the included studies. Reviewer 1 (AAAF) identified papers based on the data and resolved any disagreements over any included studies, whereas Reviewer 2 (MHMH) independently assessed abstracts and titles twice. After extensive research by reviewers to determine if the publications provided the pertinent data for the systematic review, they were selected for inclusion based on the inclusion and exclusion criteria (Table 2).

**Table 2:** Inclusion and exclusion criteria

Questions elements	Inclusion criteria	Exclusion criteria
Population	Studies involving adults (≥18 years) at risk of or with prediabetes, but without existing type 2 diabetes	Studies involving participants with established type 2 diabetes or other types of diabetes (e.g., type 1 diabetes, gestational diabetes)
Intervention	Studies assessing the use of vitamin D supplementation (any form, dose, or regimen) as a primary or adjunct intervention	Studies focusing on dietary intake of vitamin D through food rather than supplementation
Comparator	Studies comparing vitamin D supplementation with placebo, no treatment, or alternative interventions	Studies evaluating vitamin D in combination with other pharmacological interventions, where the effect of vitamin D cannot be isolated
Outcomes	Studies reporting outcomes related to the risk, prevention, or onset of type 2 diabetes (e.g., incidence, biomarkers of glucose metabolism, insulin sensitivity)	Studies that do not directly assess outcomes related to type 2 diabetes risk or prevention
Study Design	Randomized controlled trials (RCTs), cohort studies, or case-control studies	Editorials, commentaries, conference abstracts, reviews, or meta-analyses
Study language	Studies published in English	Studies published in any other language

Microsoft® Excel Spreadsheet (Microsoft, Inc., Redmond, Wash., USA) was used to collect the data from included studies.

Risk-of-Bias Assessment

The risk of bias in the included studies was assessed using the Newcastle-Ottawa Scale (NOS). Studies were categorized as low, moderate, or high based on selection process bias, intervention bias, departure from intervention bias, missing data bias, outcome bias, and result bias. The inclusion and exclusion criteria were used to establish a preference for selection. Performance bias was assessed by providing a control arm and accounting for allocation concealment. Different rankings were given to data management, biased reporting, selective reporting, and full industrial sponsorship. Over several sessions, reviewers looked at eligibility limitations and reporting consistency. When a second reviewer chose the research, any discrepancies in the reviewers' rankings were taken into account.

Results

Search Results

**Figure 1:**
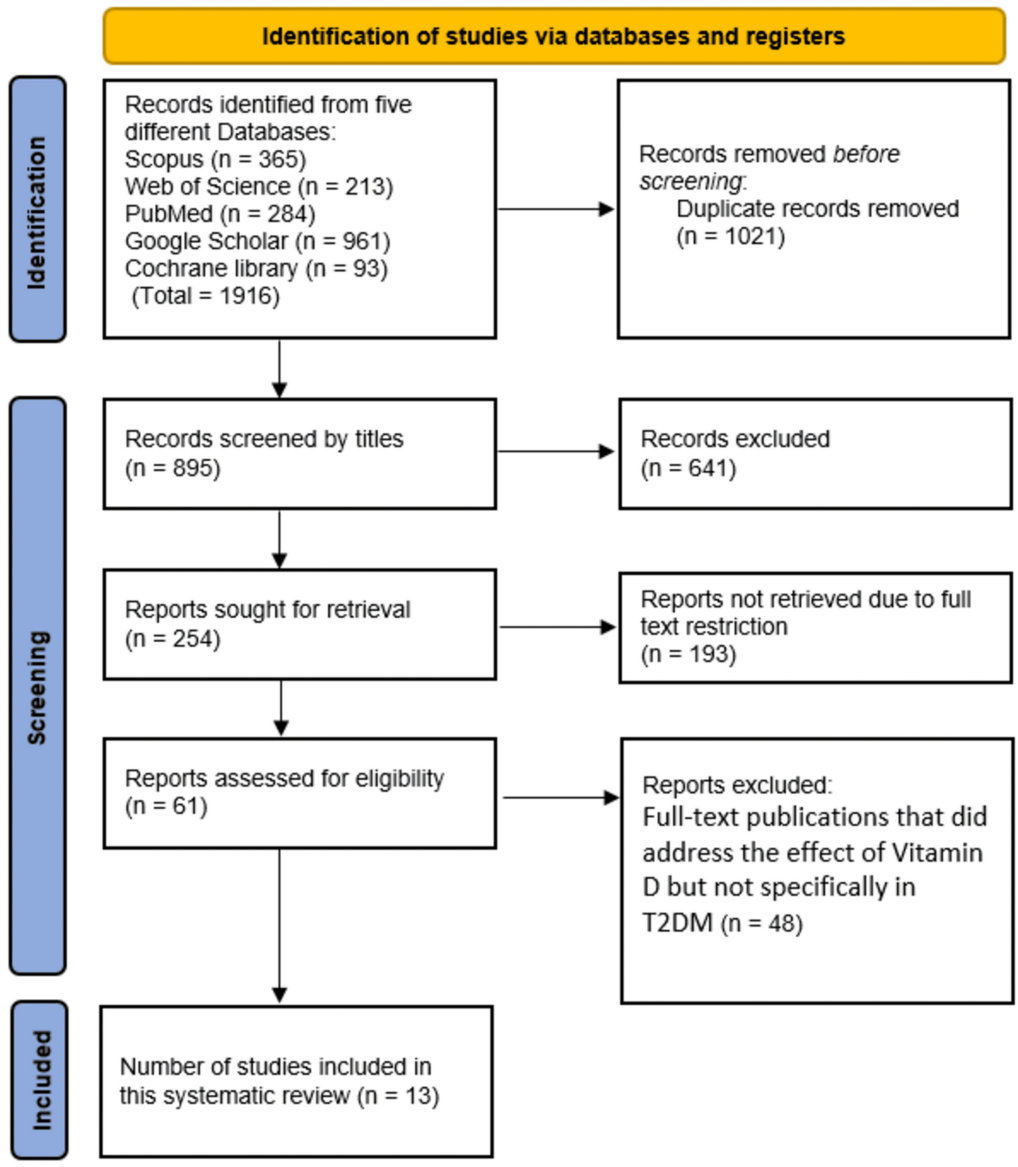
Preferred Reporting Items for Systemic Reviews and Meta-Analyses (PRISMA) flowchart

We found 1,916 studies that met the study selection criteria, and 1,021 of them were removed as duplicate records. After evaluating 895 studies based on their titles, 641 were deemed unnecessary and eliminated. After searching the remaining 254 studies to see if the complete texts of the papers were available, 193 were not found and were not included in the analysis. After 61 full-text papers were evaluated for eligibility, 48 were disqualified because they either did not directly address hypertension or telehealth strategies. This systematic review includes 13 studies that were deemed eligible (Figure 1).

Risk-of-Bias Assessment

A risk-of-bias assessment was done using the NOS. Out of 42 studies, 14 studies were found to have low-risk bias, 27 had moderate-risk bias, and 1 study had high-risk bias. In some studies, a portion of their methodological flaw is the way they chose their controls. Furthermore, no study disclosed the blinding of controls and patients concerning exposure, which may have led to measurement bias.

GRADEpro GDT indicated that the studies that were part of this meta-analysis had low quality of evidence. The inclusion of observational studies, which increases the risk of bias because it is unable to randomize the exposure, and the inconsistent nature of the research were the main causes of the low quality of the evidence (Table 3).

**Table 3:** Risk-of-bias assessment in the studies included in the systematic review using the Newcastle-Ottawa Scale (NOS) for case-control studies. Rating scale: seven to nine stars = low risk of bias; four to six stars = moderate risk of bias; 0 to three stars = high risk of bias *Selection*: (1) Is the definition adequate? (2) Is the case representativeness okay? (3) Control selection (community or hospital). (4) Control definitions. *Comparability:* (1) Comparability of controls and cases according to the analysis or design. *Exposure: *(1) Exposure determination. (2) The same method for calculation controls and cases. (3) Non-response rate. A single star (★) can be awarded to a study for each numbered item in the exhibit and selection categories. For comparability, no more than two stars (★★) can be given. *Hyphen* (-) is given in empty cells which indicates no star has been awarded to the study.

Study	Selection				Comparability	Exposure		
	1.	2.	3.	4.	1.	1.	2.	3.
Sollid et al. [13]	★	★	-	-	★★	★	-	★
Kawahara et al. [14]	★	★	-	-	★	★	★	-
Ahmed et al. [15]	★	★	-	-	-	★	★	★
Forouhi et al. [16]	★	★	★	-	★★	★	★	★
Wallace et al. [17]	★	★	-	-	★★	★	★	★
Rasouli et al. [18]	★	★	-	-	★	★	★	★
Oosterwerff et al. [19]	★	★	-	-	★★	-	★	★
Moreira‐Lucas et al. [20]	★	★	-	★	★★	★	★	★
Al Thani et al. [21]	★	★	★	-	★	-	★	★
Salehpour et al. [22]	★	★	-	-	★★	★	★	★
Jorde et al. [23]	★	★	-	-	-	-	★	-
Dutta et al. [24]	★	★	-	-	-	★	★	★
Pittas et al. [25]	★	★	★	-	★★	★	★	★

Characteristics of the Included Studies

The included studies in this systematic review collectively offer a comprehensive overview of the impact of vitamin D supplementation on the risk of developing type 2 diabetes in individuals with prediabetes. The majority of the studies employed randomized controlled trial designs, ensuring robust methodologies for evaluating outcomes. Sample sizes varied significantly, ranging from as few as 66 participants to over 2,400, allowing for a diverse range of findings across different populations and study durations. Study lengths spanned from three months to five years, providing insights into both short-term and long-term effects of vitamin D supplementation (Table 4).

**Table 4:** Characteristics and key findings of the included studies

Author	Publishing year	Study design	Sample size	Study duration	Baseline diabetes status	Vitamin D dose	Key findings
Sollid et al., [13]	2014	RCT	511	1 Year	Prediabetes	Vit D3 20,000 IU/week	The study demonstrates that in individuals with IFG and/or IGT, vitamin D supplementation had no positive effects on lipid status, blood pressure, or glycemic indices.
Kawahara et al., [14]	2022	Double-blinded, multicenter, randomized, placebo-controlled trial	1256	3 Year	Prediabetes	Eldecalcitol 30 IU/day	The results indicated that eldecalcitol may have a positive effect on those with inadequate insulin production, even while treatment with the drug did not significantly lower the incidence of Type II diabetes among those with pre-diabetes.
Ahmed et al., [15]	2020	RCT	120	3 months	Prediabetes	Vit D3 60,000 IU/week	The significance of vitamin D in glucose homeostasis was demonstrated by the enhanced insulin sensitivity observed by the OGIS index 120 minutes after the repair of hypovitaminosis D in prediabetic patients.
Forouhi et al., [16]	2016	Double-blind placebo-controlled randomized trial	340	4 months	Prediabetes	Vit D2 100,000 IU/month	HbA1c was unaffected by short-term vitamin D2 or D3 treatment. The slight decrease in PWV observed with both D2 and D3 in comparison to a placebo implies that vitamin D treatment improves arterial stiffness.
Wallace et al., [17]	2019	RCT	66	6 months	Prediabetes	Vit D3 100,000 IU/month	The study focused on a high-risk group with low 25(OH)D status at baseline and used a reliable evaluation of IR and β-cell activity. It was discovered that supplemental vitamin D3 did not affect insulin action in prediabetic individuals.
Rasouli et al., [18]	2022	RCT	1774	2 years	Prediabetes	Vit D3 3000 IU/day	Vitamin D3 supplementation for 24 months failed to enhance an OGTT-derived measure of β-cell function in prediabetes patients who were not chosen based on their baseline vitamin D status, but it did help those with extremely low baseline vitamin D levels.
Oosterwerff et al., [19]	2014	Randomized, placebo-controlled trial	130	4 months	Prediabetes	Vit D3 4000 IU/day	Supplementing with vitamin D did not alter the incidence of metabolic syndrome or increase insulin sensitivity or β cell activity in non-Western vitamin D-deficient immigrants with prediabetes. However, those who achieved a 25(OH)D concentration ≥60 nmol/L showed an improvement in the insulinogenic index when diabetes respondents were excluded.
Moreira‐Lucas et al., [20]	2017	Double-blind, randomized, placebo-controlled multicentre trial	71	6 months	Prediabetes (55.6%)	Vit D3 1200 IU/day	Oral glucose tolerance and glycaemic status indicators were not improved by weekly doses of vitamin D3 in those with poor vitamin D levels who were at risk for type 2 diabetes.
Al Thani et al., [21]	2019	RCT	209	6 months	Prediabetes	Vit D3 28,000 IU/week	Insulin sensitivity and glucose tolerance were unaffected by vitamin D administration in a cohort that was pre-diabetic and significantly vitamin deficient. It is possible that factors other than supplementing are responsible for the observed decrease in β-cell activity in both the vitamin D and placebo groups.
Salehpour et al., [22]	2013	Double-blind randomized placebo-controlled clinical trial	77	3 months	Overweight	Vit D3 30,000 IU/week	The findings show that among healthy overweight women, a 25 lg/day vitamin D3 supplement had no positive impact on glycaemic indicators.
Jorde et al., [23]	2016	RCT	511	5 years	Prediabetes	Vit D3 60,000 IU/week	Vitamin D treatment is unlikely to stop the progression of prediabetes to diabetes in individuals who do not have a vitamin D deficiency. To demonstrate such a theoretical benefit, very large trials involving people with vitamin D deficiency will likely be required.
Dutta et al., [24]	2014	RCT	170	2.5 years	Prediabetes	Vit D3 4000 IU/day	Vitamin D supplemental intake is linked to a significantly lower risk of developing diabetes (6/55 vs 13/49; p = 0.04) and a higher rate of reversal to glucose levels (23/55 vs. 10/49; p = 0.02) in people with vitamin-D insufficient or deficient prediabetes. It is also linked to a decrease in resistance to insulin and inflammation throughout the body (TNFa and IL6). Diabetes development was independently predicted by baseline vitamin D and 2-hour blood glucose.
Pittas et al., [25]	2019	RCT	2423	4 years	Prediabetes	Vit D3 60,000 IU/week	Supplementing with vitamin D3 at a level of 4000 IU daily did not significantly reduce the incidence of type 2 diabetes in people at high risk who were not chosen for vitamin D deficiency.

The baseline diabetes status across all studies was predominantly prediabetes, enabling a focused examination of this high-risk group. Various forms of vitamin D were utilized, including vitamin D2 and D3, administered in doses that ranged from 30 IU/day to 60,000 IU/week. This variation in dosing strategies facilitated the exploration of dose-response relationships and highlighted differences in efficacy depending on supplementation levels.

Key findings demonstrated a complex and often inconsistent relationship between vitamin D supplementation and diabetes-related outcomes. Several studies, such as those by Sollid et al. (2014) and Wallace et al. (2019), reported no significant improvement in glycemic indices, insulin action, or glucose tolerance despite supplementation. Similarly, investigations by Kawahara et al. (2022) and Rasouli et al. (2022) indicated that vitamin D might have limited utility in preventing diabetes progression, particularly when baseline vitamin D status was not a criterion for participant selection.

Conversely, certain studies identified potential benefits under specific conditions. Ahmed et al. (2020) observed enhanced insulin sensitivity after correcting hypovitaminosis D in prediabetic individuals, while Dutta et al. (2014) reported reduced diabetes risk and improved glycemic control in participants with low baseline vitamin D levels. Moreover, the study by Pittas et al. (2019) underscored the challenges in demonstrating a preventive effect of vitamin D supplementation, suggesting the need for larger trials targeting vitamin D-deficient populations.

Notably, several investigations highlighted the importance of baseline vitamin D levels as a determinant of supplementation efficacy. For example, Oosterwerff et al. (2014) observed improvements in insulinogenic indices among individuals achieving higher post-supplementation vitamin D concentrations. These findings emphasize the heterogeneity of responses to vitamin D supplementation, which may be influenced by baseline deficiency, dosage, and individual metabolic profiles.

Discussion

Numerous studies indicate a correlation between the occurrence of diabetes, vitamin D levels, and its impact on blood glucose. There is no solid proof that taking vitamin D supplements lowers the incidence of type 2 diabetes, as demonstrated by the RCTs examined in this review. However, in certain studies, there appears to be a relationship between levels of vitamin D and the level of insulin sensitivity.

Similar results were found in larger studies. Vitamin D levels were assessed in 1,884 cohort individuals with a four- to 11-year follow-up and 628 participants who developed diabetes as part of the Melbourne Collaborative Cohort Study. It was discovered that the chance of acquiring type 2 diabetes increased with decreasing vitamin D levels and that a 24% decreased risk of developing T2DM was linked to every 25 nmol/L increase in 25(OH)D [26]. According to a different analysis by Pittas et al. on numerous clinical trials and observational studies, the risk decrease from three sizable trials was comparable [27]. It demonstrated that those with prediabetes who did not have a vitamin D deficit had a 12% lower risk of acquiring diabetes. In one of the studies (the D2d research), additional subgroup analysis revealed that taking vitamin D supplements instead of a placebo reduced the risk of developing diabetes among individuals with a low level of vitamin D (less than 12 ng/mL) by almost 62%. Furthermore, 48% of them are thought to have converted to euglycemia. In addition, He et al. conducted a thorough study that considered the impact of vitamin D intake in several RCTs [28]. In 21 studies, vitamin D supplementation reduced fasting plasma glucose levels in pre-diabetic participants with a BMI below 25 compared to placebo, and vitamin D was most effective in lowering FPG in individuals with 25(OH)D levels from 20 to 30 ng/mL. According to 13 studies included studies, participants with baseline 25(OH)D levels higher than 30 ng/mL showed a significant improvement in insulin sensitivity. Importantly, compared to controls, vitamin D supplements of more than 2,000 IU/day resulted in a 16% decrease in the risk of type 2 diabetes in a subgroup evaluation of six studies [26-28].

Even though there is strong evidence linking vitamin D levels to the risk of type 2 diabetes, some people draw different conclusions. After an average of 7.3 years of follow-up, roughly 6.3% of postmenopausal women with vitamin D deficiency from a variety of racial backgrounds who got vitamin D went on to acquire type 2 diabetes. After controlling for a number of risk factors, there was no correlation found in this study between levels of vitamin D and the occurrence of type 2 diabetes [29]. There was no statistically significant difference in vitamin D levels among those who developed type 2 diabetes and those who did not, according to a different study by Qi et al. that included 138 out of 596 participants who developed the condition. The results remained constant even after controlling for confounders [30]. Finally, subsequent genetic studies revealed no correlation between the likelihood of developing type 2 diabetes in a population of European heritage and a genetic predisposition resulting from elevated 25(OH)D levels. Vitamin D did not have a genetic association with type 2 diabetes or any other glycemic indicators [31].

Results from evaluating how vitamin D affected several blood glucose indicators in vitamin D-deficient individuals from varied origins were consistent. Vitamin D shortage (<50 nmol/L) in overweight prediabetic adults had little effect on glycemic parameters when compared to a placebo. The difference between the groups was 0.2 mmol/L for FPG, 0.4 mmol/mol for HbA1c, and 0.2 for HOMA-IR [17]. A moderate amount of vitamin D supplemented after four months did not significantly alter impaired fasting glucose levels or impaired glucose tolerance when compared to placebo in non-Western individuals with high prediabetes, BMI, and a lack of vitamin D in the Netherlands [19]. Post-intervention HOMA-IR and HbA1c values were similar even after controlling for age, sex, and BMI. Pre-diabetic Qatari participants with vitamin D levels below 30 ng/mL showed comparable outcomes. After a six-month intervention, the vitamin D group's mean change on the two-hour sugar tolerance test was 1.15 against 1.09 (mmol/L) for the placebo group [21]. Another small-scale double-blind RCT involving a 24-week monitoring revealed no discernible difference among the group receiving vitamin D or placebo groups, who were pre-diabetic with primarily 25(OH)D < 50 nmol/L, in terms of the main outcome of a two-hour 75 g test for oral glucose tolerance after vitamin D intervention. In the latter two investigations, even secondary results were not particularly significant [20]. In addition, the change in HbA1c was negligible for participants with vitamin D deficiency, which was roughly 50% to 58% of those over the 50 nmol/L limit in both the control and treatment groups with prediabetic risk. The decrease was -0.05% for those receiving vitamin D2 and 0.02% for those receiving vitamin D3 compared to placebo [16].

Conflicting results were found in other trials involving subjects who were not impaired. Following a 12-week course of vitamin D supplemental use, there was almost no statistically or biochemically significant difference between the vitamin D group and placebo in terms of HOMA-IR, HbA1c, and two-hour postprandial hyperglycemia in healthy yet obese women. The vitamin D group had higher levels of glucose than the placebo group, even when it came to FPG. However, there was a link between Hba1c and serum vitamin D levels, and average FPG and HBa1c levels dropped in both groups [22, 32]. Similarly, when comparing groups to their baseline, there was very little improvement in β-cell activity and sensitivity to insulin in prediabetic participants with high BMI. After 24 months, there was an average difference of 3.7% less in the group taking vitamin D and 2.2% less in the placebo group [18]. However, certain groups of those with vitamin D levels below 12 ng/mL in the untreated group experienced a change of 4.9%, whereas those in the placebo group experienced a decrease of -13.6%.

The degree to which vitamin D supplementation affected patients' incidence of type 2 diabetes and their insulin sensitivity differed from research to study in this systematic analysis. The 13 trials that were considered showed a notable degree of variation in the type of vitamin D supplement utilized, primary and secondary consequences, participant characteristics, and study duration. Healthy patients who have adequate vitamin D levels, due to higher levels of exercise, better diets, and more exposure to sunlight, may also have lower levels of insulin resistance than less healthy individuals who lead more sedentary lives, eat less healthily, get less sunlight, and have lower vitamin D levels.

Limitations

The study data varied throughout the trials in a number of ways, including sample size, baseline vitamin D level, participant ethnicity, and additional vitamin D dosage. Furthermore, the majority of trials did not account for variables that affect levels of vitamin D, such as exposure to sunlight, degree of physical activity, and renal function. Similar to this, the majority of clinical trials did not take into account variables that affect the resistance to insulin, such as family history, physical activity level, and food. Although the major goal of this comprehensive review was to determine how vitamin D supplementation affected the incidence of type 2 diabetes, six out of the 13 RCTs that were found did not list type 2 diabetes incidence as a primary or secondary outcome.

Recommendations and future directions

Future studies should include more massive, methodologically sound RCTs targeting population subgroups with deficiencies in vitamin D status at baseline to investigate the preventive impact of supplementation on T2DM. Further research should seek to examine how best to dose, for how long oxygen should be supplied, and if it interacts with other metabolic parameters in a way that identifies the subgroups that could benefit most. Clinicians treating patients and policymakers are advised to study the patient’s major risk factors and the baseline vitamin D status of the patients due to the ineffective and ineffectual one-size-fits-all plans for supplementing to protect against the formation of diabetes.

## Conclusions

Overall, there is not enough data from our comprehensive review to conclude that taking vitamin D supplements considerably postpones the onset of type 2 diabetes. However, in a few trials, vitamin D supplementation was observed to affect the level of insulin resistance in subjects. RCTs will probably be necessary to see whether this can prevent the development of type 2 diabetes. In order to impact clinical recommendations for vitamin D supplementation in individuals with insulin resistance, further RCTs with comparable vitamin D dosages may assist in elucidating the connection between vitamin D and T2DM.
